# Draft Genome Sequence of *Enterobacter* sp. AS-1, a Potential Eurytrophic Recombination Host

**DOI:** 10.7150/jgen.53040

**Published:** 2021-01-01

**Authors:** Yuki Iwasaki, Yuya Itoiri, Sota Ihara, Hironaga Akita, Mamoru Oshiki, Zen-ichiro Kimura

**Affiliations:** 1 National Institute of Technology (KOSEN), Kure College (NIT, Kure), 2-2-11, Agaminami, Kure, Hiroshima 737-8506, Japan; 2 Research Institute for Sustainable Chemistry, National Institute of Advanced Industrial Science and Technology (AIST), 3-11-32, Kagamiyama, Higashi-hiroshima, Hiroshima 739-0046, Japan; 3 Division of Environmental Engineering, Faculty of Engineering, Hokkaido University, North 13, West 8, Sapporo, Hokkaido, 060-8628, Japan

**Keywords:** *Enterobacter*, Eurytrophic, Recombination host, Whole genome sequence

## Abstract

Strain AS-1 was isolated from laboratory-scale activated sludge collected in Japan.

This strain not only grows on rich medium, including R2A medium, but also forms colonies on medium lacking organic matter other than agar (water agar), indicating it could be used as a eurytrophic recombinant host in material production processes. Here, we present a draft genome sequence of *Enterobacter* sp. AS-1, which consists of a total of 24 contigs containing 5,207,146 bp, with a GC content of 55.64%, and comprising 4,921 predicted coding sequences. Based on 16S rRNA gene sequence analysis, strain AS-1 was designated as *Enterobacter* sp. AS-1.

## Introduction

Current processes for producing fuels, chemicals, and other valuable products are often catalyzed by genetically modified organisms. The use of genetically modified organisms makes it possible to design processes that would not be possible using wild-type strains 1. In many cases, the ability to produce a substance depends on the host organism's inherent physiologic properties and material-production capacity, and therefore, host performance is often a bottleneck in production process design 2. *Escherichia coli*, widely used as a recombinant prokaryotic host organism, is a good example demonstrating the ease of cultivation, recombination methodologies, and suitability of the recombinant host for use in the design of production processes 3. *Escherichia coli* presents numerous advantages in this regard, including ease of establishing host strains. In addition to the properties mentioned above, we conceived of 'eurytrophic' as a beneficial host trait. The process of producing valuable resources using eurytrophic bacteria is similar to that of using *E. coli* in eutrophic cultures but has a lower cost than eutrophic cultures. In addition, the process is stable under poor nutritional conditions, thus reducing the risk of gene mutations 4. Another advantage of using eurytrophic bacteria is that contamination during handling of the fermentation reactor can be prevented by using broad nutrient availability as the selective pressure. These properties suggest that there is a great deal of merit in using broad-spectrum bacteria as hosts. Here, we report the successful isolation of a recombinant host of an extremely poorly nourished bacterium capable of growing on medium (water agar) free of organic matter except for a gelation agent such as agar, targeting the *Enterobacteriaceae* family of *E. coli*. Genome sequencing was performed for future detailed genomic studies and industrial applications of this strain.

## Materials and Methods

Laboratory-scale activated sludge was collected from Kure city in Hiroshima Prefecture, Japan. Water agar plates (containing only agar [Wako, Japan]) were used for strain isolation. The plates were inoculated with 0.1 mL each of a 0.01% (v/v) dilution of sample solution and incubated for 5 days at 25°C. Thereafter, single colonies were re-streaked onto new R2A plates at least three times to obtain pure colonies. Water agar plates and R2A plates were used to select strains exhibiting eurytrophic properties. The resulting isolated strains were cultivated in R2A broth, and the cells for electroporation were collected by centrifugation and washed twice with sterile water.

The suitability of the isolated strains as recombinant hosts was assessed based on their ability to express the pUC19 plasmid and exhibit ampicillin resistance 5. The cyclically tuned pUC19 plasmid (10 nmol) was introduced into washed cells by electroporation (Gene Pulser II, Bio-Rad, Hercules, CA), and introduced cells were verified based on the ability of the bacteria to form colonies on R2A + ampicillin medium. One strain (AS-1) capable of expressing the ampicillin resistance gene encoded in this plasmid was acquired.

We next determined a draft genome sequence for strain AS-1. A sample was prepared for genome sequencing by growing strain AS-1 aerobically overnight at 25 °C in R2A medium (Daigo, Japan). Genomic DNA was then extracted from the culture using a DNeasy Plant Mini kit (Qiagen, Hilden, Germany) according to the manufacturer's instructions. The concentration and purity of the genomic DNA were determined using a NanoDrop ND-1000 spectrophotometer (Thermo Fisher Scientific, Waltham, MA, USA) and a Quant-iT dsDNA BR assay kit (Invitrogen, Waltham, MA, USA). The genomic DNA was sequenced using MinION (Oxford Nanopore Technologies, Oxford, UK; flow cell version R9 and rapid sequencing kit) and MiSeq (Illumina, San Diego, CA, USA) sequencers. Default parameters were used for all software unless otherwise specified. Hybrid de novo assembly of the raw data was carried out using Unicycler, ver. 0.4.7 6. Genome annotation was performed using DFAST ver.1.2.4 7. 16S rRNA gene sequences of the related type strains were compared with reference sequences available in the GenBank/EMBL/DDBJ databases using BLAST. Construction of a maximum-likelihood tree was performed using FastTree2 8, 9.

## Results and Discussion

Diluted activated sludge was inoculated onto water agar medium, which was free of organic matter except for agar, and incubated at room temperature for 5 days, resulting in the isolation of 12 colonies. The isolates were further tested for their ability to grow on R2A medium, and the most rapidly growing strains were selected. This manipulation was used to screen potential eurytrophic recombination hosts. To further validate the suitability of strain AS-1 as a host, the pUC19 plasmid was introduced by electroporation, and the ability to express the ampicillin resistance gene encoded in this plasmid was tested. The results showed that strain AS-1 introducing pUC19 could grow on R2A + ampicillin agar. In addition, pUC19 plasmid was extracted from the transformed strain, and the AS-1 wild-type strain became resistant to ampicillin by reintroduction of this plasmid. Thus, the AS-1 strain was able to express the ampicillin resistance gene encoded by the pUC19 plasmid. Which led us to conclude that strain AS-1 could be used as a eurytrophic recombinant host.

We next determined a draft genome sequence for strain AS-1. The raw data from the MinION and Miseq analyses included 1,102,715 and 2,344,370 reads, respectively, with 574.9-coverage. The genome sequence was 5,205,692 bp, and the GC content was 55.64%. The assembly generated 18 contigs, with an N50 contig size of 2,915,782 bp. A total of 5,013 predicted coding sequences were identified. In addition, 86 tRNA genes and 22 rRNA genes were detected using DFAST ver.1.2.4. To determine the phylogeny of strain AS-1, a maximum-likelihood tree based on 16S rRNA gene sequences was constructed. In the resultant phylogenetic tree, strain AS-1 fell inside the cluster comprising members of the genus *Enterobacter*.

Moreover, strain AS-1 exhibited similarities of 99.8%, 99.7%, 99.5%, and 99.3% to its closest relatives, *E. sichuanensis* WCHECL1597^T^ 10, *E. chengduensis* WCHECl-C4^T^ 11, *E. wuhouensis* WCHEs120002^T^ 12, and *E. chuandaensis* 90028^T^ 13, respectively. Thus, strain AS-1 was designated as *Enterobacter* sp. AS-1. A comparison of the genomic features of *Enterobacter* sp. AS-1, *E. sichuanensis* WCHECL1597^T^, *E. chengduensis* WCHECl-C4T, *E. wuhouensis* WCHEs120002^T^, and *E. chuandaensis* 90028^T^ revealed similar numbers of tRNA and rRNA genes as well as similar GC content (Table 1). These results will be described in more detail elsewhere as the next stage of our study.

### Nucleotide Sequence Accession Numbers

The draft genome sequence of strain AS-1 was deposited in the DDBJ/EMBL/GenBank databases under accession numbers BMBZ01000001 to BMBZ01000018. The raw sequence reads were deposited in the DDBJ under BioProject number PRJDB10378 and BioSample number SAMD00239574.

## Figures and Tables

**Figure 1:**
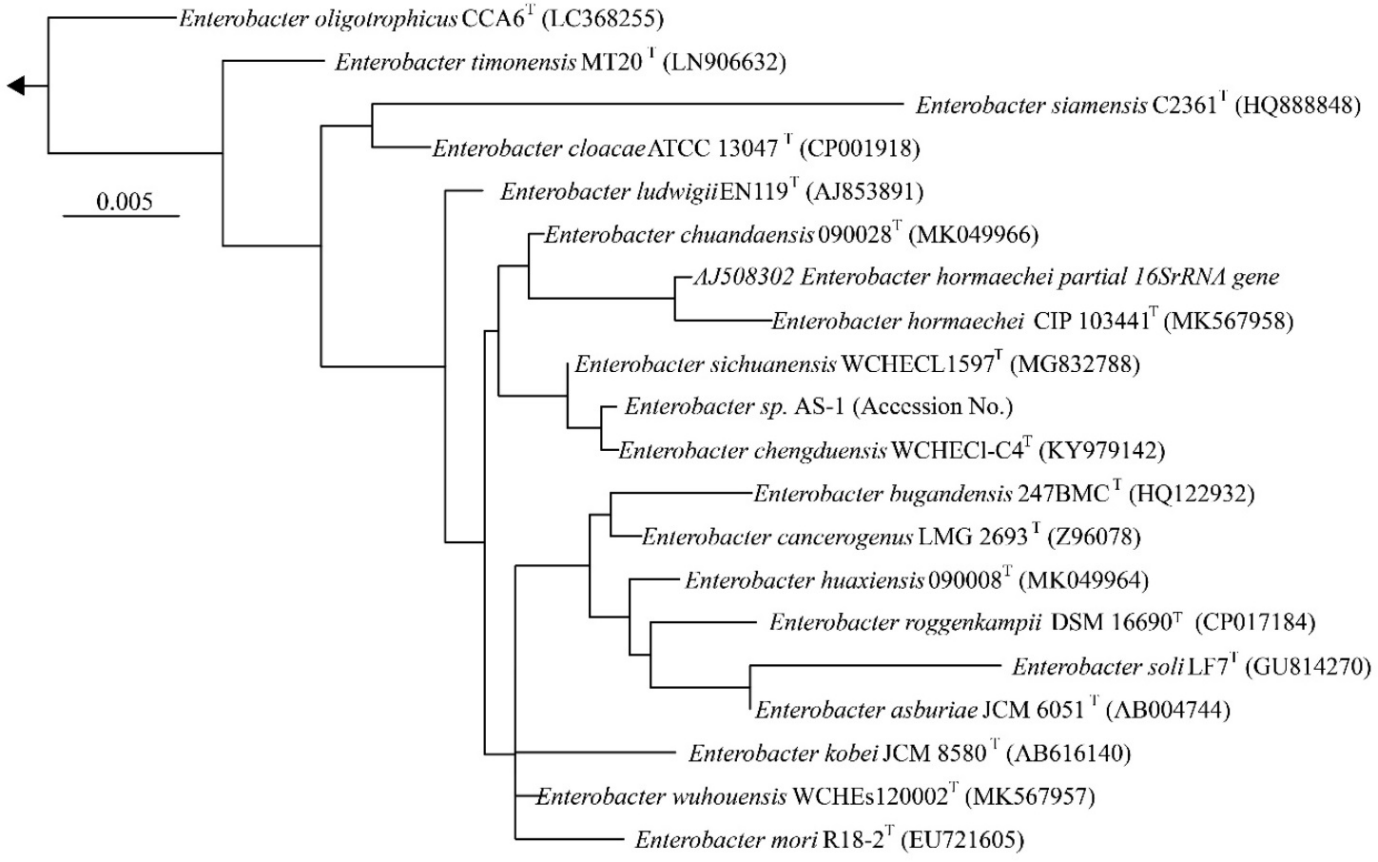
Phylogenetic tree constructed from analysis of 16S rRNA gene sequences showing the relationships between strain AS-1 and its related type strains. The bar indicates a 0.005% nucleotide substitution rate. The arrows are directed to the outgroup, E. coli.

**Table 1:** Comparison of general genome features of strain AS-1 with the related type strains.

Strain	Strain AS-1	*E. sichuanensis* WCHECL1597^T^ (MG832788)	*E. chengduensis* WCHECl-C4^T^ (KY979142)	*E. wuhouensis* WCHEs120002^T^ (MK567957)	*E. chuandaensis* 90028^T^ (MK049966)
Properties					
Genome length (bp)	5,205,692	4,897,201	3,264,334	4,897,369	3,236,984
GC content (%)	55.6	55.2	70.6	56.1	70.2
Contig numbers	22	204	1	46	1
Coding sequence numbers	5,013	4,501	3,049	4,690	2,944
tRNA	86	40	49	75	49
rRNA	22	3	9	3	9
